# An Efficient Multi-Path Multitarget Tracking Algorithm for Over-The-Horizon Radar †

**DOI:** 10.3390/s19061384

**Published:** 2019-03-20

**Authors:** Yuan Huang, Yifang Shi, Taek Lyul Song

**Affiliations:** 1 Department of Electronic Systems Engineering, Hanyang University, Ansan 15588, Korea; hy4335657@hotmail.com (Y.H.); tsong@hanyang.ac.kr (T.L.S.); 2 School of Automation, Hangzhou Dianzi University, Xiasha Higher Education Zone, Hangzhou 310018, China

**Keywords:** multitarget tracking, linear multitarget process, data association, OTHR, multiple detection, target existence evaluation

## Abstract

In target tracking environments using over-the-horizon radar (OTHR), one target may generate multiple detections through different signal propagation paths. Trackers need to jointly handle the uncertainties stemming from both measurement origin and measurement path. Traditional multitarget tracking algorithms suffer from high computational loads in such environments since they need to enumerate all possible joint measurement-to-track assignments considering the measurements paths unless they employ some approximations regarding the measurements and their corresponding paths. In this paper, we propose a novel algorithm, named multi-path linear multitarget integrated probabilistic data association (MP-LM-IPDA), to efficiently track multitarget in multiple detection environments. Instead of generating all possible joint assignments, MP-LM-IPDA calculates the modulated clutter measurement density for each measurement cell of each track. The modulated clutter measurement density considers the possibility that the measurement cells originate from the clutter as well as from other potential targets. By incorporating the modulated clutter measurement density, the single target tracking structure can be applied for multitarget tracking, which significantly reduces the computational load. The simulation results demonstrate the effectiveness and efficiency of the proposed algorithm.

## 1. Introduction

Multitarget tracking is a challenging problem that requires confirming potential targets as well as estimating target states. The most widely used tracking algorithms, including multiple hypothesis tracker (MHT) [1], joint probabilistic data association (JPDA) [2] and algorithms based on the random finite set (RFS) theory [3,4,5], have been shown to be effective with specific models and assumptions in various scenarios.

The above-mentioned algorithms have a common assumption that one target can generate at most one measurement at each scan time, which is the single detection assumption. However, in many practical scenarios, one target may produce more than one measurement per scan. A well-known example of such multiple detection (MD) systems is over-the-horizon radar (OTHR) [6,7]. In the over-the-horizon radar (OTHR) system, the detection signals are reflected off ionospheric layers to detect the area beyond the radar horizon for long-range targets surveillance. Radar signals from the same target arrive at the receiver via different signal propagation paths, resulting in multiple detections of one target. The multiple detection mechanism in the OTHR system brings benefits and problems to tracking algorithms at the same time. Compared with traditional radars, the primary advantage of OTHR is that it can detect and track over-the-horizon targets, generating a great deal of interest in both military and civilian groups. Meanwhile, the main challenge in OTHR is multi-path signal propagation, which requires the tracking algorithm to jointly handle the uncertainties from both the measurement origin and the measurement path.

There are two widely used methods for multiple detection pattern, one is the measurement partition method [8] and the other is the random matrix method [9,10]. In the OTHR system, only the measurement partition method is suitable. The random matrix assumes extended target or group targets, however, the target in the OTHR system is still treated as a point target. In OTHR systems, the measurement partition method is utilized to generate possible target detection sets, where each set contains one or some of the selected measurements. Then, non-repeated paths are assigned to the measurements of a set to take the measurement path uncertainty into consideration. The path combined measurement sets are used by trackers for both true track confirmation and target state estimatation through various data association structures. Multiple detection structures outperform conventional single detection algorithms when applied to OTHR systems due to the sufficient utilization of target information from different paths. However, the complexity of the multiple detection structure exponentially increases in accordance with increases in the numbers of tracks and possible sets of target generated measurements. More serious is the path uncertainty of each measurement aggravates the computational burden.

Recently, several algorithms have been designed that explicitly consider the multiple-detection pattern. Such an algorithm of multiple detection JPDA (MD-JPDA) is proposed in [11] for OTHR-based multitarget tracking. In MD-JPDA, the association probabilities are calculated based on the probabilistic inference made on no measurement or a measurement set originated from a target. In [12], the authors proposed a track splitting structure for multitarget tracking in the OTHR system, and the proposed algorithm had a better true track confirmation performance than other approaches. In multiple detection multitarget tracking, the above-mentioned two algorithms are cumbersome due to numerous joint events used to assign measurements to tracks. In [13], a multiple detection multiple hypothesis tracker (MD-MHT) is proposed, in which the structure of the track hypothesis tree is very complex. More recent algorithms include the multiple detection probability hypothesis density filter [14,15] and multi-path Bernoulli filter [16,17], which are designed for the OTHR system based on random finite set theory.

Due to the high computational cost, traditional joint data association structures are suitable for scenarios in which only a small number of targets cross each other. To jointly consider the uncertainties in the measurement origin and measurement path of the OTHR-based system, this paper proposes multi-path linear multitarget integrated probabilistic data association (MP-LM-IPDA) for efficient multitarget tracking. The linear multitarget integrated probabilistic data association (LM-IPDA) algorithm [18,19] is designed for multitarget tracking, which bypasses joint measurement-to-track assignments utilizing the modulated clutter measurement density. The modulated clutter measurement density evaluates the possibility that a measurement is generated from clutter as well as other potential targets. Thus, the data association events are generated for each track separately as if they are for single target tracking, and the computational complexity linearly increases with the numbers of tracks and measurements. LM-IPDA is the basis of our proposed algorithm.

In MP-LM-IPDA, measurement cells consisting of one or more selected measurements are generated after the measurement selection step. The path pattern, which is a set of ordered measurement paths, is combined with a measurement cell to ensure that each measurement of the measurement cell has a path indicating the model from which a measurement is generated. Then, the modulated clutter measurement density for each path pattern combined measurement cell is evaluated. Multiple detection joint data association structures generate joint track-to-measurement cell assignments among tracks and measurement cells. However, the MP-LM-IPDA algorithm generates track-to-measurement cell associations for a track τ separately by considering the influence of other tracks on track τ through the measurement cell in terms of the modulated clutter measurement density. The modulated clutter measurement density is used in the track update step with a little addition of computational cost.

As contributions of this paper, the following statement can be made: In the OTHR system, the multiple detection issue with the measurement path uncertainties significantly aggravate computational loads of multitarget tracking algorithms. This is due to the fact that the path uncertainty and the multiple detection issue lead to a three dimensional data association. To realize an efficient tracker for this special tracking system, the multi-path version of the LM-IPDA algorithm is proposed in this paper. Almost every multitarget tracking algorithm for OTHR employs a certain form of approximation to reduce or limit the number of track-to-measurement association events or hypotheses. However, the MP-LM-IPDA algorithm entirely bypasses the explicit joint track-to-measurement assignment step. Without joint track-to-measurement assignments, each track of MP-LM-IPDA is propagated using a multi-path single target tracking structure with the modulated clutter measurement density. The algorithm proposed in this paper is different from MD-LM-IPDA proposed in [20], which only considers the measurement origin uncertainty for multiple detection multitarget tracking using a standard radar. This paper is an extension of the conference paper [21], which is the forerunner that demonstrates the most important results of MP-LM-IPDA.

This paper is organized as follows. Section 2 discusses the assumptions and models for target tracking using OTHR. The detailed derivations of MP-LM-IPDA are demonstrated in Section 3. Simulation studies and conclusions are given in Section 4 and Section 5, respectively.

## 2. Assumptions and Models

The assumptions and models used for target tracking in the OTHR system are provided in this section. Targets are located at a very long range that is beyond the horizon of the radar system. To detect potential targets, the high-frequency wave first reflects through ionospheric layers and then reaches the targets. Different ionospheric layers create multi-paths for the detecting signal, resulting in transmitted signals scattered by the target that arrive at the receiver via different propagation paths. A target can be detected through each path with a certain detection probability (usually less than unity), which leads to multiple detections from one target. In multitarget tracking using OTHR, the correspondence among the target, the measurement origin and the signal propagation path is unknown to the tracker.

**Figure 1:**
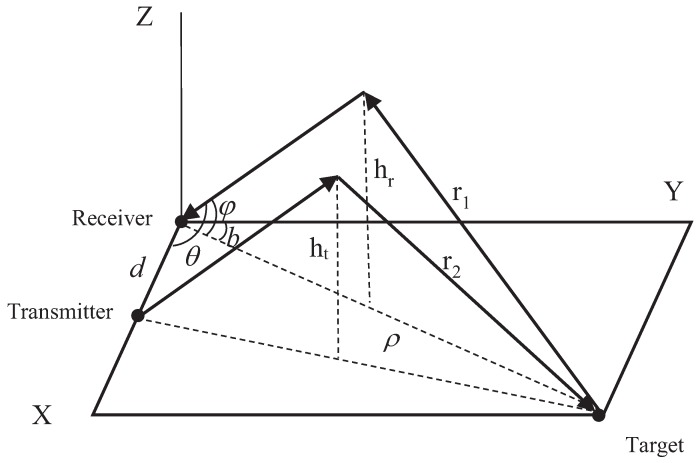
Geometry of the planar OTHR system.

Key assumptions: (1) The Earth is assumed as flat, i.e., the planar OTHR measurement geometry of Figure 1 is used in this paper. In this planar geometry, the target motion is in the same plane. The planar OTHR geometry can be easily changed to a spherical geometry [22] with modifications considering the curvature of the Earth. (2) The heights of the ionospheric layers are assumed to be known and fixed, i.e., each ionospheric layer has a constant layer hight. In [23,24,25], the ionospheric heights are assumed to be unknown and then jointly estimated with the target state. (3) For simplicity, there are two ionospheric layers considered in this paper, i.e., E-layer and F-layer, with constant heights vertical heights of $h_{E}$ and $h_{F}$, respectively. These key assumptions are widely used in the researches for the OTHR system [6,11,14].

### 2.1. Target Motion Model

The target sate $x_{k}^{\tau }$ discussed in this paper consists of the ground range, ground range rate, bearing and bearing rate $x_{k}^{\tau }={\left[\rho ,\dot{\rho },b,\dot{b}\right]}^{T}$. The target motion is confined to the X-Y plane, as shown in Figure 1. The discrete-time form nearly constant velocity (NCV) model is used for target state propagation, which is

(1) $$x_{k+1}^{\tau }=Fx_{k}^{\tau }+v_{k}^{\tau },$$

where $x_{k+1}^{\tau }$ is the state of target τ at scan k+1, *F* is the propagation matrix, and $v_{k}^{\tau }$ represents the process noise between scan *k* and k+1, which has a zero-mean Gaussian pdf with covariance *Q*.

### 2.2. Measurement Generation Model

In Figure 1, the receiver is at the origin, with the transmitter situated on the X-axis at a distance *d* from it. The Z-axis is the vertical direction. The distance between the target and the receiver is defined as the ground range ρ, and the angle with respect to boresight is defined as the bearing *b*. Idealized reflecting ionospheric layers are present at heights $h_{t}$ (of the transmit layer) and $h_{r}$ (of the receive layer). Half of the slant ranges from the target to the receiver and from the transmitter to the target are denoted by $r_{1}$ and $r_{2}$, respectively.

**Figure 2:**
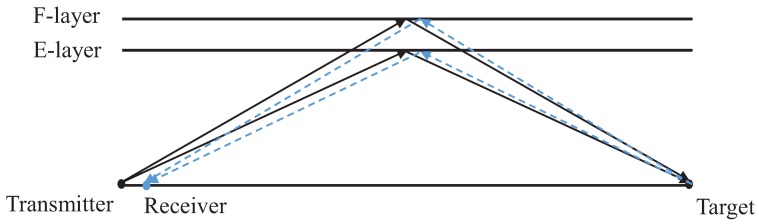
Radar signal propagates through different ionospheric layers.

A two-layer signal propagation geometry is depicted in Figure 2, in which the *E*-layer and *F*-layer are assumed to have vertical heights of $h_{E}$ and $h_{F}$, respectively. With two ionospheric layers (*E* and *F*), there are four possible signal propagation paths, as shown in Table 1.

In Table 1, there are four possible signal propagation paths, each represented by a known measurement generation function. Denoting $z_{i}\left(k\right)$ as the *i*th measurement at scan *k*, the path-dependent measurement generation model can be written as

(2) $$\begin{array}{c} z_{i}\left(k\right)=\left\{\begin{array}{cc} \begin{array}{c} h_{EE}\left(x_{k}\right)+w_{EE,k}\\ h_{EF}\left(x_{k}\right)+w_{EF,k}\\ h_{FE}\left(x_{k}\right)+w_{FE,k}\\ h_{FF}\left(x_{k}\right)+w_{FF,k}\\ clutter \end{array} & \begin{array}{c} \mathrm{model}\;\;\;\mathrm{EE},\;\;\;\mathrm{Pat}h\;1\\ \mathrm{model}\;\;\;\mathrm{EF},\;\;\;\mathrm{Pat}h\;2\\ \mathrm{model}\;\;\;\mathrm{FE},\;\;\;\mathrm{Pat}h\;3\\ \mathrm{model}\;\;\;\mathrm{FF},\;\;\;\mathrm{Pat}h\;4\\ \mathrm{otherwise}, \end{array} \end{array}\right. \end{array}$$

where $h_{tr}$ ($t,r\in \left\{E,F\right\}$) represents the measurement generation function with respect to a specific signal propagation path with transmit layer *t* and receive layer *r*. The measurement noise terms $w_{tr,k}$ are mutually independent, follow a zero-mean Gaussian distribution with covariance *R*, and are uncorrelated with $v_{k}^{\tau }$.

The measurement from the OTHR consists of the slant range, the rate of change of the slant range, and the apparent azimuth $\left[R_{g},R_{r},A_{z}\right]$ [6], calculated by

(3) Rg=r1+r2Rr=ρ˙4ρr1+ηr2Az=sin−1ρsinbρsinb2r12r1,

where

(4) r1=r1ρ,hr=Δρρ222+hr2r2=r2ρ,b,hr=Δρρ222−dρsinbdρsinb22+dd222+ht2η=ρ−dsinb.

For simplicity, the detection probabilities of different paths are set to be the same, given by

(5) $$P_{DEE}=P_{DEF}=P_{DFE}=P_{DFF}=P_{D}.$$

Clutter measurements also arise at each scan. The uniform/Poisson model, consisting of a uniform spatial pdf for each clutter measurement and a Poisson probability mass function for the number of clutter measurements, is used to generate clutter measurements in this paper. Clutter measurements are assumed to be independent of each other.

At each scan, each track utilizes the gating method [26] to select measurements. The set of all selected measurements at scan *k* is represented by $Z_{k}$ and consists of both target measurements and clutter measurements, given by

(6) $$Z_{k}={\left\{z_{i}\left(k\right)\right\}}_{i=1}^{m_{k}},$$

where $m_{k}$ is the total number of selected measurements at scan *k*.

The cumulative set of measurements collected from the initial to current scan is $Z^{k}$, given by

(7) $$Z^{k}=\left\{Z_{1},Z_{2},\ldots ,Z_{k}\right\}.$$

At each scan, the measurements selected by a track are used to estimate the target state and evaluate the target existence probability under the multi-path pattern.

## 3. Multi-Path Linear Multitarget Integrated Probabilistic Data Association

This section introduces the detailed derivations of MP-LM-IPDA. We begin in Section 3.1 with a single target tracking structure for the OTHR system called multi-path integrated probabilistic data association (MP-IPDA). Then, the notion of the modulated clutter measurement density is introduced, which is the core of applying the single target tracking structure to the multitarget tracking. Finally, we obtain MP-LM-IPDA, where evaluations of data associations and target existences are based on the modulated clutter measurement density in place of the pure clutter measurement density.

### 3.1. Track State Expression

During tracking, tracks are initialized and updated using the selected measurements. These selected measurements can be target detections or false alarms, which leads to tracks that may track targets (which are treated as true tracks) or clutter (which are treated as false tracks). The true or false status of a track is evaluated according to the tracking performance, which is mainly based on the data association results. In this paper, the track state at scan *k* consists of the trajectory state and the target existence event. The track state is expressed by a hybrid pdf, given as

(8) $$p\left[x_{k}^{\tau },\chi_{k}^{\tau }|Z^{k}\right]=p\left(x_{k}^{\tau }|\chi_{k}^{\tau },Z^{k}\right)P\left(\chi_{k}^{\tau }|Z^{k}\right),$$

where the trajectory state is defined only for a given target existence.

In this manuscript, $p\left(x_{k}^{\tau }|\chi_{k}^{\tau },Z^{k}\right)$ and $P\left(\chi_{k}^{\tau }|Z^{k}\right)$ are propagated according to the following predict-update manner [27]:

(9) $$\begin{array}{c} p\left(x_{k-1}^{\tau }|\chi_{k-1}^{\tau },Z^{k-1}\right)\\ P\left(\chi_{k-1}^{\tau }|Z^{k-1}\right) \end{array}\to \begin{array}{c} p\left(x_{k}^{\tau }|\chi_{k}^{\tau },Z^{k-1}\right)\\ P\left(\chi_{k}^{\tau }|Z^{k-1}\right) \end{array}\to \begin{array}{c} data\\ association \end{array}\to \begin{array}{c} p\left(x_{k}^{\tau }|\chi_{k}^{\tau },Z^{k}\right)\\ P\left(\chi_{k}^{\tau }|Z^{k}\right) \end{array}$$

The main difference among various tracking algorithms lies in the data association mechanism, i.e., how to build measurement-to-track relations and how to evaluate corresponding association probabilities.

### 3.2. Measurement Utilization

Since the multi-path problem is considered in this paper, the measurements selected by a track are first used to generate measurement cells, and then these measurement cells are combined with suitable paths. The path combined measurement cells are used for the data association to update the track state. Each measurement cell is a set of possible target detections, consisting of one or some of the selected measurements. Combining a measurement cell with a proper path ensures that each measurement in the measurement cell has a non-repeated measurement generation model.

For example, assume that four measurements $\left\{z_{1}\left(k\right),z_{2}\left(k\right),z_{3}\left(k\right),z_{4}\left(k\right)\right\}$ are selected by track τ and that there are four paths, as shown in Table 1. Then, measurement cells are generated as follows:

- Assuming that only one of the selected measurements is generated by the target ($\varphi_{\tau }=1$, i.e., each measurement cell consists of one of the selected measurements), and the corresponding measurement cells are
  $$\begin{array}{c} \begin{array}{c} z_{1,1}\left(k\right)=\left\{z_{1}\left(k\right)\right\},z_{1,2}\left(k\right)=\left\{z_{2}\left(k\right)\right\},z_{1,3}\left(k\right)=\left\{z_{3}\left(k\right)\right\},z_{1,4}\left(k\right)=\left\{z_{4}\left(k\right)\right\}. \end{array} \end{array}$$
  then the measurement cell-related parameters are $c_{1}=C_{1}^{4}=4$ and $n_{1}\in \left\{1,2,3,4\right\}$ in this case.
- Assuming that two of the selected measurements are generated by the target ($\varphi_{\tau }=2$, i.e., each measurement cell consists of two of the selected measurements), and the corresponding measurement cells are
  $$\begin{array}{c} \begin{array}{c} \begin{array}{c} z_{2,1}\left(k\right)=\left\{z_{1}\left(k\right),z_{2}\left(k\right)\right\},z_{2,2}\left(k\right)=\left\{z_{1}\left(k\right),z_{3}\left(k\right)\right\},z_{2,3}\left(k\right)=\left\{z_{1}\left(k\right),z_{4}\left(k\right)\right\},\\ z_{2,4}\left(k\right)=\left\{z_{2}\left(k\right),z_{3}\left(k\right)\right\},z_{2,5}\left(k\right)=\left\{z_{2}\left(k\right),z_{4}\left(k\right)\right\},z_{2,6}\left(k\right)=\left\{z_{3}\left(k\right),z_{4}\left(k\right)\right\}. \end{array} \end{array} \end{array}$$
  then $c_{2}=C_{2}^{4}=6$ and $n_{2}\in \left\{1,2,3,4,5,6\right\}$.
- Assuming that three of the selected measurements are generated by the target ($\varphi_{\tau }=3$, i.e., each measurement cell consists of three of the selected measurements), and the corresponding measurement cells are
  $$\begin{array}{c} \begin{array}{c} \begin{array}{c} z_{3,1}\left(k\right)=\left\{z_{1}\left(k\right),z_{2}\left(k\right),z_{3}\left(k\right)\right\},z_{3,2}\left(k\right)=\left\{z_{1}\left(k\right),z_{2}\left(k\right),z_{4}\left(k\right)\right\},\\ z_{3,3}\left(k\right)=\left\{z_{1}\left(k\right),z_{3}\left(k\right),z_{4}\left(k\right)\right\},z_{3,4}\left(k\right)=\left\{z_{2}\left(k\right),z_{3}\left(k\right),z_{4}\left(k\right)\right\}. \end{array} \end{array} \end{array}$$
  then $c_{3}=C_{3}^{4}=4$ and $n_{3}\in \left\{1,2,3,4\right\}$.
- Assuming that all of the selected measurements are generated by the target ($\varphi_{\tau }=4$, i.e., a measurement cell consists of all of the selected measurements), and the corresponding measurement cell is
  $$\begin{array}{c} \begin{array}{c} z_{4,1}\left(k\right)=\left\{z_{1}\left(k\right),z_{2}\left(k\right),z_{3}\left(k\right),z_{4}\left(k\right)\right\}. \end{array} \end{array}$$
  then $c_{4}=C_{4}^{4}=1$ and $n_{4}\in \left\{1\right\}$. After the measurement cell generation, a total of 15 measurement cells are formed using the selected measurements under different assumptions on the number of target-originated measurements.

In the OTHR system, different signal propagating paths correspond to different measurement generation models. Therefore, the measurement cell path pattern should be assigned to each measurement cell to utilize the measurement cells. The measurement cell path pattern contains the path for each measurement of a specified measurement cell. Denote by

(10) $$A_{j}\left(z_{\varphi_{\tau },n_{\varphi_{\tau }}}\left(k\right)\right),j=\left\{1,2,\ldots ,\frac{L!}{\left(L-\varphi_{\tau }\right)!}\right\}$$

the measurement cell $z_{\varphi_{\tau },n_{\varphi_{\tau }}}\left(k\right)$ that is generated by a path pattern $A_{j}\left(z_{\varphi_{\tau },n_{\varphi_{\tau }}}\left(k\right)\right)$. $A_{j}\left(z_{\varphi_{\tau },n_{\varphi_{\tau }}}\left(k\right)\right)$ represents that $z_{\varphi ,n_{\varphi }}\left(k\right)$ originates from one of $\frac{L!}{\left(L-\varphi \right)!}$ possible path pattern allocations. For example, suppose that L=4 for the measurement cell $z_{2,2}\left(k\right)=\left\{z_{1}\left(k\right),z_{3}\left(k\right)\right\}$, then the the set of possible measurement cell path patterns is

(11) $$\begin{array}{c} \begin{array}{c} \begin{array}{c} \left\{\left(1,2\right),\left(2,1\right),\left(1,3\right),\left(3,1\right),\left(1,4\right),\left(4,1\right),\left(2,3\right),\left(3,2\right),\left(2,4\right),\left(4,2\right),\left(3,4\right),\left(4,3\right)\right\}. \end{array} \end{array} \end{array}$$

For example, the measurement cell path pattern can be chosen as $A_{5}\left(z_{2,2}\left(k\right)\right)=\left(1,4\right)$, representing that measurement $z_{1}\left(k\right)$ is generated through path 1 and measurement $z_{3}\left(k\right)$ is generated through path 4.

In MP-LM-IPDA, the likelihoods of the path combined measurement cells are used for calculating the modulated clutter measurement density. The truncated pdf of $z_{\varphi_{\tau },n_{\varphi_{\tau }}}\left(k\right)$ through path pattern $A_{j}\left(z_{\varphi_{\tau },n_{\varphi_{\tau }}}\left(k\right)\right)$ is restricted inside the validation gate of track τ, satisfying

(12) $$\begin{array}{c} p\left(\langle z_{\varphi_{\tau },n_{\varphi_{\tau }}}\left(k\right)|A_{j}\left(z_{\varphi_{\tau },n_{\varphi_{\tau }}}\left(k\right)\right)\rangle |\chi_{k}^{\tau },Z^{k-1}\right)=\frac{N\left(z_{\varphi_{\tau },n_{\varphi_{\tau }}}\left(k\right)|\zeta_{A_{j}\left(z_{\varphi_{\tau },n_{\varphi_{\tau }}}\left(k\right)\right)},S_{A_{j}\left(z_{\varphi_{\tau },n_{\varphi_{\tau }}}\left(k\right)\right)}\right)}{{\left(P_{G}\right)}^{\varphi_{\tau }}}. \end{array}$$

In Equation (12), the Gaussian pdf is used for measurement cell $z_{\varphi_{\tau },n_{\varphi_{\tau }}}\left(k\right)$ allocated with the measurement cell path pattern $A_{j}\left(z_{\varphi_{\tau },n_{\varphi_{\tau }}}\left(k\right)\right)$. $P_{G}$ is the gating probability of a single detection through a path. $\zeta_{A_{j}\left(z_{\varphi_{\tau },n_{\varphi_{\tau }}}\left(k\right)\right)}$ represents the measurement prediction based on the track state prediction with respect to the measurement cell path pattern $A_{j}\left(z_{\varphi_{\tau },n_{\varphi_{\tau }}}\left(k\right)\right)$. $S_{A_{j}}\left(z_{\varphi_{\tau },n_{\varphi_{\tau }}}\left(k\right)\right)$ is the corresponding measurement cell innovation covariance with respect to measurement cell path pattern $A_{j}\left(z_{\varphi_{\tau },n_{\varphi_{\tau }}}\left(k\right)\right)$. The details of calculating the parameters used in Equation (12) can be found in [6,7].

### 3.3. MP-IPDA

The single target tracking structure of MP-IPDA is used for the track update in MP-LM-IPDA. The probabilities of data associations and target existence for the measurement update step of MP-IPDA are developed in this section. The obtained expressions of MP-IPDA are straightforward modifications of IPDA [28], with differences arising from the multi-path pattern leading to a non-homogeneous clutter environment.

For an established track τ, the number of path pattern combined measurement cells is *M*. A total of M+1 possible data association events are considered for the track, such that one of the path pattern combined measurement cells is from the potential target or all the path pattern combined measurement cells are from clutter. The a posteriori track state estimate under each data association event is generated, along with the corresponding data association probability. The contribution of each path pattern combined measurement cell to the overall a posteriori track state estimate is weighted according to the data association probability.

The a posteriori probability that $z_{\varphi_{\tau },n_{\varphi_{\tau }}}\left(k\right)$ is generated by target τ through path pattern $A_{j}\left(z_{\varphi_{\tau },n_{\varphi_{\tau }}}\left(k\right)\right)$ is given by

(13) $$\begin{array}{cc} \beta_{k,\langle z_{\varphi_{\tau },n_{\varphi_{\tau }}}\left(k\right)|A_{j}\left(z_{\varphi_{\tau },n_{\varphi_{\tau }}}\left(k\right)\right)\rangle }^{\tau }= & P\left(\chi_{k,\langle z_{\varphi_{\tau },n_{\varphi_{\tau }}}\left(k\right)|A_{j}\left(z_{\varphi_{\tau },n_{\varphi_{\tau }}}\left(k\right)\right)\rangle }^{\tau }|\chi_{k}^{\tau },Z^{k}\right)\\ = & \frac{P\left(\chi_{k,\langle z_{\varphi_{\tau },n_{\varphi_{\tau }}}\left(k\right)|A_{j}\left(z_{\varphi_{\tau },n_{\varphi_{\tau }}}\left(k\right)\right)\rangle }^{\tau },\chi_{k}^{\tau }|Z^{k}\right)}{P\left(\chi_{k}^{\tau }|Z^{k}\right)}. \end{array}$$

Similarly, the a posteriori probability that all the measurements are from clutter given target τ exists is calculated by

(14) $$\beta_{k,0}^{\tau }=P\left(\chi_{k,0}^{\tau }|\chi_{k}^{\tau },Z^{k}\right)=\frac{P\left(\chi_{k,0}^{\tau },\chi_{k}^{\tau }|Z^{k}\right)}{P\left(\chi_{k}^{\tau }|Z^{k}\right)}.$$

The numerator of Equation (13) is written out explicitly, using Bayes’ formula, as

(15) $$\begin{array}{c} \begin{array}{c} \begin{array}{c} P\left(\chi_{k,\langle z_{\varphi_{\tau },n_{\varphi_{\tau }}}\left(k\right)|A_{j}\left(z_{\varphi_{\tau },n_{\varphi_{\tau }}}\left(k\right)\right)\rangle }^{\tau },\chi_{k}^{\tau }|Z^{k}\right)\\ =c_{k}^{-1}p\left(Z_{k}|\chi_{k,\langle z_{\varphi_{\tau },n_{\varphi_{\tau }}}\left(k\right)|A_{j}\left(z_{\varphi_{\tau },n_{\varphi_{\tau }}}\left(k\right)\right)\rangle }^{\tau },\chi_{k}^{\tau },Z^{k-1}\right)P\left(\chi_{k,\langle z_{\varphi_{\tau },n_{\varphi_{\tau }}}\left(k\right)|A_{j}\left(z_{\varphi_{\tau },n_{\varphi_{\tau }}}\left(k\right)\right)\rangle }^{\tau }|\chi_{k}^{\tau },Z^{k-1}\right)\bar{\psi } \end{array}, \end{array} \end{array}$$

where $c_{k}$ is a normalization constant.

The joint density of measurement set $Z_{k}$, given that measurement cell $z_{\varphi_{\tau },n_{\varphi_{\tau }}}\left(k\right)$ is generated by target τ through path pattern $A_{j}\left(z_{\varphi_{\tau },n_{\varphi_{\tau }}}\left(k\right)\right)$ and all other measurements in $Z_{k}$ are clutter measurements, is the product of probability density functions of the path pattern combined measurement cells and clutter measurements, which is

(16) $$\begin{array}{c} \begin{array}{c} \begin{array}{c} p\left(Z_{k}|\chi_{k,\langle z_{\varphi_{\tau },n_{\varphi_{\tau }}}\left(k\right)|A_{j}\left(z_{\varphi_{\tau },n_{\varphi_{\tau }}}\left(k\right)\right)\rangle }^{\tau },\chi_{k}^{\tau },Z^{k-1}\right)\\ =p\left(\langle z_{\varphi_{\tau },n_{\varphi_{\tau }}}\left(k\right)|A_{j}\left(z_{\varphi_{\tau },n_{\varphi_{\tau }}}\left(k\right)\right)\rangle |\chi_{k}^{\tau },Z^{k-1}\right)p_{k}^{c}\left(Z_{k}|\chi_{k,\langle z_{\varphi_{\tau },n_{\varphi_{\tau }}}\left(k\right)|A_{j}\left(z_{\varphi_{\tau },n_{\varphi_{\tau }}}\left(k\right)\right)\rangle }^{\tau },\chi_{k}^{\tau },Z^{k-1}\right), \end{array} \end{array} \end{array}$$

where $p\left(\langle z_{\varphi_{\tau },n_{\varphi_{\tau }}}\left(k\right)|A_{j}\left(z_{\varphi_{\tau },n_{\varphi_{\tau }}}\left(k\right)\right)\rangle |\chi_{k}^{\tau },Z^{k-1}\right)$ is given in Equation (12).

The clutter likelihood $p_{k}^{c}\left(Z_{k}|\chi_{k,0}^{\tau },\chi_{k}^{\tau },Z^{k-1}\right)$, given that all the measurements of $Z_{k}$ are clutter, satisfies the non-homogeneous Poisson distribution [29], given by

(17) $$p_{k}^{c}\left(Z_{k}|\chi_{k,0}^{\tau },\chi_{k}^{\tau },Z^{k-1}\right)=\mu_{F}\left(m_{k}\right)\prod_{i=1}^{m_{k}}\frac{\rho_{z_{i}\left(k\right)}^{\tau }}{\lambda },$$

where $\lambda =\int_{V}\rho_{k,z}dz$ is the mean number of non-homogeneous clutter measurements in the validation gate with volume *V*. The probability density of observation $z_{i}\left(k\right)$ is ρzikτρzikτλλ, and the probability of observing $m_{k}$ measurements is $\mu_{F}\left(m_{k}\right)=\frac{\lambda^{m_{k}}}{m_{k}!}e^{-\lambda }$, which follows a Poisson distribution.

Therefore, $p_{k}^{c}\left(Z_{k}|\chi_{k,\langle z_{\varphi_{\tau },n_{\varphi_{\tau }}}\left(k\right)|A_{j}\left(z_{\varphi_{\tau },n_{\varphi_{\tau }}}\left(k\right)\right)\rangle }^{\tau },\chi_{k}^{\tau },Z^{k-1}\right)$ in Equation (16) can be calculated based on Equation (17) by eliminating the measurements in $z_{\varphi_{\tau },n_{\varphi_{\tau }}}\left(k\right)$ from $Z_{k}$, given by

(18) $$\begin{array}{c} p_{k}^{c}\left(Z_{k}|\chi_{k,\langle z_{\varphi_{\tau },n_{\varphi_{\tau }}}\left(k\right)|A_{j}\left(z_{\varphi_{\tau },n_{\varphi_{\tau }}}\left(k\right)\right)\rangle }^{\tau },\chi_{k}^{\tau },Z^{k-1}\right)=\mu_{F}\left(m_{k}-\varphi_{\tau }\right)\prod_{\forall z_{i}\left(k\right)\notin z_{\varphi_{\tau },n_{\varphi_{\tau }}}\left(k\right)}\frac{\rho_{z_{i}\left(k\right)}^{\tau }}{\lambda }. \end{array}$$

The a priori probability $P\left(\chi_{k,\langle z_{\varphi_{\tau },n_{\varphi_{\tau }}}\left(k\right)|A_{j}\left(z_{\varphi_{\tau },n_{\varphi_{\tau }}}\left(k\right)\right)\rangle }^{\tau }|\chi_{k}^{\tau },Z^{k-1}\right)$ in Equation (15) satisfies

(19) $$\begin{array}{c} P\left(\chi_{k,\langle z_{\varphi_{\tau },n_{\varphi_{\tau }}}\left(k\right)|A_{j}\left(z_{\varphi_{\tau },n_{\varphi_{\tau }}}\left(k\right)\right)\rangle }^{\tau }|\chi_{k}^{\tau },Z^{k-1}\right)\\ =\frac{L!}{\varphi_{\tau }!\left(L-\varphi_{\tau }\right)!}{\left(P_{D}P_{G}\right)}^{\varphi_{\tau }}{\left(1-P_{D}P_{G}\right)}^{L-\varphi_{\tau }}\frac{\varphi_{\tau }!\left(m_{k}-\varphi_{\tau }\right)!}{m_{k}!}\frac{\left(L-\varphi_{\tau }\right)!}{L!}, \end{array}$$

which consists of detecting and selecting $\varphi_{\tau }$ measurements with a specific path.

In Equation (15), $\bar{\psi }$ is the notation for $P\left(\chi_{k}^{\tau }|Z^{k-1}\right)$, which is the predicted probability of target existence and is calculated by

(20) $$\begin{array}{c} \begin{array}{c} P\left(\chi_{k}^{\tau }|Z^{k-1}\right)=a_{11}P\left(\chi_{k-1}^{\tau }|Z^{k-1}\right)+a_{21}P\left({\bar{\chi }}_{k-1}^{\tau }|Z^{k-1}\right), \end{array} \end{array}$$

where $P\left(\chi_{k-1}^{\tau }|Z^{k-1}\right)$ and $P\left({\bar{\chi }}_{k-1}^{\tau }|Z^{k-1}\right)$ are the updated probabilities of target existence and target non-existence at scan k−1, respectively. $a_{11}$ and $a_{21}$ are the transition probabilities that describe the change of existence state between scans, defined as

(21) $$a_{11}=P\left(\chi_{k}^{\tau }|\chi_{k-1}^{\tau }\right),$$

(22) $$a_{21}=P\left(\chi_{k}^{\tau }|{\bar{\chi }}_{k-1}^{\tau }\right).$$

The target existence and non-existence probabilities satisfy

(23) $$P\left(\chi_{k}^{\tau }|Z^{l}\right)+P\left({\bar{\chi }}_{k}^{\tau }|Z^{l}\right)=1,\left(l\in \left\{k-1,k\right\}\right).$$

Combining Equations (16), (19) and (20) into Equation (15) yields $P\left(\chi_{k,\langle z_{\varphi_{\tau },n_{\varphi_{\tau }}}\left(k\right)|A_{j}\left(z_{\varphi_{\tau },n_{\varphi_{\tau }}}\left(k\right)\right)\rangle }^{\tau },\chi_{k}^{\tau }|Z^{k}\right)$.

Using the theorem of total probability, we have

(24) $$P\left(\chi_{k,0}^{\tau }|Z^{k}\right)+\sum_{\varphi_{\tau }=1}^{\varphi_{\tau ,\max}}\sum_{n_{\varphi_{\tau }}=1}^{c_{\varphi_{\tau }}}\sum_{j}P\left(\chi_{k,\langle z_{\varphi_{\tau },n_{\varphi_{\tau }}}\left(k\right)|A_{j}\left(z_{\varphi_{\tau },n_{\varphi_{\tau }}}\left(k\right)\right)\rangle }^{\tau },\chi_{k}^{\tau }|Z^{k}\right)=1,$$

in which the event $\left\{\chi_{k,0}^{\tau }\right\}$ (no measurement is originated from target τ) is the union of event $\left\{\chi_{k,0}^{\tau },\chi_{k}^{\tau }\right\}$ (no measurement is originated from target τ and target τ exists) and event $\left\{\chi_{k,0}^{\tau },{\bar{\chi }}_{k}^{\tau }\right\}$ (no measurement originates from target τ and target τ does not exist). Thus, the probability of $\left\{\chi_{k,0}^{\tau }\right\}$ is

(25) $$P\left(\chi_{k,0}^{\tau }|Z^{k}\right)=P\left(\chi_{k,0}^{\tau },\chi_{k}^{\tau }|Z^{k}\right)+P\left(\chi_{k,0}^{\tau },{\bar{\chi }}_{k}^{\tau }|Z^{k}\right).$$

$P\left(\chi_{k,0}^{\tau },\chi_{k}^{\tau }|Z^{k}\right)$ in Equation (25) is calculated by

(26) $$\begin{array}{cc} P\left(\chi_{k,0}^{\tau },\chi_{k}^{\tau }|Z^{k}\right)= & c_{k}^{-1}p_{k}^{c}\left(Z_{k}|\chi_{k,0}^{\tau },\chi_{k}^{\tau },Z^{k-1}\right)P\left(\chi_{k,0}^{\tau }|\chi_{k}^{\tau },Z^{k-1}\right)\bar{\psi }\\ = & c_{k}^{-1}p_{k}^{c}\left(Z_{k}|\chi_{k,0}^{\tau },\chi_{k}^{\tau },Z^{k-1}\right)\left(1-P_{Dec}^{\tau }\right)\bar{\psi }\\ = & c_{k}^{-1}\frac{e^{-\lambda }}{m_{k}!}\prod_{i=1}^{m_{k}}\rho_{z_{i}\left(k\right)}^{\tau }\left(1-P_{Dec}^{\tau }\right)\bar{\psi }, \end{array}$$

where Equation (17) is used for $p_{k}^{c}\left(Z_{k}|\chi_{k,0}^{\tau },\chi_{k}^{\tau },Z^{k-1}\right)$, and $P_{Dec}^{\tau }$ is the probability that there is at least one target τ generated measurement among the $m_{k}$ gated measurements for the multi-path pattern such that

(27) $$P_{Dec}^{\tau }=\sum_{\varphi_{\tau }=1}^{L}P_{DG\varphi_{\tau }}^{\tau }=\sum_{\varphi_{\tau }=1}^{L}\frac{L!{\left(P_{D}P_{G}\right)}^{\varphi_{\tau }}{\left(1-P_{D}P_{G}\right)}^{L-\varphi_{\tau }}}{\varphi_{\tau }!\left(L-\varphi_{\tau }\right)!}.$$

Similar to Equation (26), $P\left(\chi_{k,0}^{\tau },{\bar{\chi }}_{k}^{\tau }|Z^{k}\right)$ in Equation (25) is

(28) $$\begin{array}{cc} P\left(\chi_{k,0}^{\tau },{\bar{\chi }}_{k}^{\tau }|Z^{k}\right)= & c_{k}^{-1}p_{k}^{c}\left(Z_{k}|\chi_{k,0}^{\tau },{\bar{\chi }}_{k}^{\tau },Z^{k-1}\right)P\left(\chi_{k,0}^{\tau }|{\bar{\chi }}_{k}^{\tau },Z^{k-1}\right)P\left({\bar{\chi }}_{k}^{\tau }|Z^{k-1}\right)\\ = & c_{k}^{-1}\frac{e^{-\lambda }}{m_{k}!}\prod_{i=1}^{m_{k}}\rho_{k,z_{i}}^{\tau }\left(1-\bar{\psi }\right). \end{array}$$

$P\left(\chi_{k,0}^{\tau }|Z^{k}\right)$ is obtained by substituting Equations (26) and (28) into Equation (25).

Through Equation (24), the normalization constant $c_{k}$ used in the calculations can be obtained by

(29) $$c_{k}=\frac{e^{-\lambda }}{m_{k}!}\prod_{i=1}^{m_{k}}\rho_{k,z_{i}}^{\tau }\left(1-\left(1-\Lambda_{k}^{\tau }\right)\bar{\psi }\right),$$

in which $\Lambda_{k}^{\tau }$ is the measurement likelihood ratio, defined as

(30) $$\begin{array}{c} \Lambda_{k}^{\tau }\overset{\Delta }{\;=}1-P_{Dec}^{\tau }+\sum_{\varphi_{\tau }=1}^{\varphi_{\tau ,\max}}\sum_{n_{\varphi_{\tau }}=1}^{c_{\varphi_{\tau }}}\sum_{j}\frac{p_{\langle z_{\varphi_{\tau },n_{\varphi_{\tau }}}\left(k\right)|A_{j}\left(z_{\varphi_{\tau },n_{\varphi_{\tau }}}\left(k\right)\right)\rangle }^{\tau }}{\rho_{\langle z_{\varphi_{\tau },n_{\varphi_{\tau }}}\left(k\right)|A_{j}\left(z_{\varphi_{\tau },n_{\varphi_{\tau }}}\left(k\right)\right)\rangle }^{\tau }}{\left(P_{D}P_{G}\right)}^{\varphi_{\tau }}{\left(1-P_{D}P_{G}\right)}^{L-\varphi_{\tau }}, \end{array}$$

where $p_{\langle z_{\varphi_{\tau },n_{\varphi_{\tau }}}\left(k\right)|A_{j}\left(z_{\varphi_{\tau },n_{\varphi_{\tau }}}\left(k\right)\right)\rangle }^{\tau }$ is defined in Equation (12), and $\rho_{\langle z_{\varphi_{\tau },n_{\varphi_{\tau }}}\left(k\right)|A_{j}\left(z_{\varphi_{\tau },n_{\varphi_{\tau }}}\left(k\right)\right)\rangle }^{\tau }$ is calculated by

(31) $$\rho_{\langle z_{\varphi_{\tau },n_{\varphi_{\tau }}}\left(k\right)|A_{j}\left(z_{\varphi_{\tau },n_{\varphi_{\tau }}}\left(k\right)\right)\rangle }^{\tau }=\prod_{\forall z_{i}\left(k\right)\in z_{\varphi_{\tau },n_{\varphi_{\tau }}}\left(k\right)}\rho_{z_{i}\left(k\right)}^{\tau },$$

where $\rho_{z_{i}\left(k\right)}^{\tau }=\rho$, $\forall z_{i}\left(k\right)\in z_{\varphi_{\tau },n_{\varphi_{\tau }}}\left(k\right)$ for independent clutter measurements.

$P\left(\chi_{k}^{\tau }|Z^{k}\right)$, which is the denominator of Equations (13) and (14), satisfies

(32) $$\begin{array}{c} \begin{array}{c} P\left(\chi_{k}^{\tau }|Z^{k}\right)=P\left(\chi_{k,0}^{\tau },\chi_{k}^{\tau }|Z^{k}\right)+\sum_{\varphi_{\tau }=1}^{\varphi_{\tau ,\max}}\sum_{n_{\varphi_{\tau }}=1}^{c_{\varphi_{\tau }}}\sum_{j}P\left(\chi_{k,\langle z_{\varphi_{\tau },n_{\varphi_{\tau }}}\left(k\right)|A_{j}\left(z_{\varphi_{\tau },n_{\varphi_{\tau }}}\left(k\right)\right)\rangle }^{\tau },\chi_{k}^{\tau }|Z^{k}\right). \end{array} \end{array}$$

The probability of target existence (PTE) is updated by Equation (32) with Equation (30), given by

(33) $$P\left(\chi_{k}^{\tau }|Z^{k}\right)=\frac{\Lambda_{k}^{\tau }P\left(\chi_{k}^{\tau }|Z^{k-1}\right)}{1-\left(1-\Lambda_{k}^{\tau }\right)P\left(\chi_{k}^{\tau }|Z^{k-1}\right)},$$

where $P\left(\chi_{k}^{\tau }|Z^{k-1}\right)$ is calculated by (20).

The data association probabilities of Equations (13) and (14) are finally obtained as

(34) $$\beta_{\langle z_{\varphi_{\tau },n_{\varphi_{\tau }}}\left(k\right)|A_{j}\left(z_{\varphi_{\tau },n_{\varphi_{\tau }}}\left(k\right)\right)\rangle }^{\tau }=\frac{{\left(P_{D}P_{G}\right)}^{\varphi_{\tau }}{\left(1-P_{D}P_{G}\right)}^{L-\varphi_{\tau }}}{\Lambda_{k}^{\tau }}\frac{p_{\langle z_{\varphi_{\tau },n_{\varphi_{\tau }}}\left(k\right)|A_{j}\left(z_{\varphi_{\tau },n_{\varphi_{\tau }}}\left(k\right)\right)\rangle }^{\tau }}{\prod_{\forall z_{i}\left(k\right)\in z_{\varphi_{\tau },n_{\varphi_{\tau }}}\left(k\right)}\rho_{k,z_{i}\left(k\right)}^{\tau }}$$

and

(35) $$\beta_{k,0}^{\tau }=\frac{1-P_{Dec}^{\tau }}{\Lambda_{k}^{\tau }},$$

respectively.

### 3.4. Modulated Clutter Measurement Density for the Path Pattern Combined Measurement Cell

In the MP-LM-IPDA algorithm, the modulated clutter measurement density forms the basis of constructing the computationally efficient multitarget tracking algorithm from the single target tracking structure, but without enumerating the feasible joint events among tracks, measurements and paths.

The modulated clutter measurement density is a modification of the pure environment clutter density by taking into account the contribution of other potential targets on a path pattern combined measurement cell. The extra calculation involved in the modulated clutter measurement density is minor, resulting in the computational load of MP-LM-IPDA linearly increasing with the numbers of tracks and path pattern combined measurement cells.

The modulated clutter measurement density for multi-path multitarget tracking with non-homogeneous clutter is developed in this section, which is used to replace the non-homogeneous clutter measurement density used in MP-IPDA.

Denote by $P_{\langle z_{\varphi_{\tau },n_{\varphi_{\tau }}}\left(k\right)|A_{j}\left(z_{\varphi_{\tau },n_{\varphi_{\tau }}}\left(k\right)\right)\rangle }^{\tau }$ the probability that target τ exists and measurement cell $z_{\varphi_{\tau },n_{\varphi_{\tau }}}\left(k\right)$ is generated by target τ through path pattern $A_{j}\left(z_{\varphi_{\tau },n_{\varphi_{\tau }}}\left(k\right)\right)$. In the LM framework, the a priori data association probabilities are calculated by first assuming that there is only one potential target. Since $z_{\varphi_{\tau },n_{\varphi_{\tau }}}\left(k\right)$ is one of the path pattern combined measurement cells that contain $\varphi_{\tau }$ detections, $P_{\langle z_{\varphi_{\tau },n_{\varphi_{\tau }}}\left(k\right)|A_{j}\left(z_{\varphi_{\tau },n_{\varphi_{\tau }}}\left(k\right)\right)\rangle }^{\tau }$ is calculated by

(36) Pzφτ,nφτk|Ajzφτ,nφτkτ=ΔPχkτ|Zk−1PDGφττpzφτ,nφτk|Ajzφτ,nφτkτpzφτ,nφτk|Ajzφτ,nφτkτρφτρφτ∑nφτ∑jpzφτ,nφτk|Ajzφτ,nφτkτpzφτ,nφτk|Ajzφτ,nφτkτρφτρφτ,

in which pzφτ,nφτk|Ajzφτ,nφτkτpzφτ,nφτk|Ajzφτ,nφτkτρφτρφτ∑nφτ∑jpzφτ,nφτk|Ajzφτ,nφτkτpzφτ,nφτk|Ajzφτ,nφτkτρφτρφτ is the ratio of exponential distances indicating the relative closeness of $z_{\varphi_{\tau },n_{\varphi_{\tau }}}\left(k\right)$ with the specified path pattern $A_{j}\left(z_{\varphi_{\tau },n_{\varphi_{\tau }}}\left(k\right)\right)$ to the predicted measurement of track τ among the measurement cells with $\varphi_{\tau }$ measurements through different path patterns. $p_{\langle z_{\varphi_{\tau },n_{\varphi_{\tau }}}\left(k\right)|A_{j}\left(z_{\varphi_{\tau },n_{\varphi_{\tau }}}\left(k\right)\right)\rangle }^{\tau }$ is the path pattern combined measurement cell likelihood defined in Equation (12), and ρ denotes the pure environment clutter density.

For the measurement cell that contains only one measurement with a specified measurement path, the probability $P_{\langle z_{i}\left(k\right)|A_{j}\left(z_{i}\left(k\right)\right)\rangle }^{\tau }$ is

(37) Pzik|Ajzikτ=ΔPχkτ|Zk−1PDG1τpzik|Ajzikτpzik|Ajzikτρρ∑n1∑jpzik|Ajzikτpzik|Ajzikτρρ,

which is a single detection version of Equation (36).

Assume that there are a total of *T* tracks under consideration in the environment at scan *k*. The probability that the path pattern combined measurement cell $\langle z_{\varphi_{\tau },n_{\varphi_{\tau }}}\left(k\right)|A_{j}\left(z_{\varphi_{\tau },n_{\varphi_{\tau }}}\left(k\right)\right)\rangle$ is not generated by any other potential targets excluding target τ is defined by

(38) $$\begin{array}{c} \begin{array}{c} Q_{\tau ,\langle z_{\varphi_{\tau },n_{\varphi_{\tau }}}\left(k\right)|A_{j}\left(z_{\varphi_{\tau },n_{\varphi_{\tau }}}\left(k\right)\right)\rangle }^{0}\overset{\Delta }{\;=}\prod_{\forall \sigma \in T\setminus \tau }\prod_{\Theta }\left(1-P_{\langle z_{i}\left(k\right)|A_{j}\left(z_{i}\left(k\right)\right)\rangle }^{\sigma }\right). \end{array} \end{array}$$

The influence on $\langle z_{\varphi_{\tau },n_{\varphi_{\tau }}}\left(k\right)|A_{j}\left(z_{\varphi_{\tau },n_{\varphi_{\tau }}}\left(k\right)\right)\rangle$ from the other potential targets excluding target τ represented in (38) is regarded as that of clutter by disassembling $\langle z_{\varphi_{\tau },n_{\varphi_{\tau }}}\left(k\right)|A_{j}\left(z_{\varphi_{\tau },n_{\varphi_{\tau }}}\left(k\right)\right)\rangle$ into path combined measurement cells consisting of single measurements. In the above calculation, Θ is notation for $\forall \langle z_{i}\left(k\right)|A_{j}\left(z_{i}\left(k\right)\right)\rangle \in \langle z_{\varphi_{\tau },n_{\varphi_{\tau }}}\left(k\right)|A_{j}\left(z_{\varphi_{\tau },n_{\varphi_{\tau }}}\left(k\right)\right)\rangle$, which represents utilizing every single measurement in $z_{\varphi_{\tau },n_{\varphi_{\tau }}}\left(k\right)$ combined with the corresponding single measurement path in $A_{j}\left(z_{\varphi_{\tau },n_{\varphi_{\tau }}}\left(k\right)\right)$. For instance, $\langle z_{2,2}\left(k\right)|\left(1,4\right)\rangle$, which, as mentioned in Section 3.2, should be disassembled into $\langle z_{1}\left(k\right)|\left(1\right)\rangle$ and $\langle z_{3}\left(k\right)|\left(4\right)\rangle$ for the calculation of (38).

If both σ and τ are omitted from the set *T*, the expression of Equation (38) is changed to

(39) $$\begin{array}{c} \begin{array}{c} Q_{\tau ,\langle z_{\varphi_{\tau },n_{\varphi_{\tau }}}\left(k\right)|A_{j}\left(z_{\varphi_{\tau },n_{\varphi_{\tau }}}\left(k\right)\right)\rangle }^{\sigma }\overset{\Delta }{\;=}\prod_{\forall w\in T\setminus \left(\tau ,\sigma \right)}\prod_{\Theta }\left(1-P_{\langle z_{i}\left(k\right)|A_{j}\left(z_{i}\left(k\right)\right)\rangle }^{w}\right)=\frac{Q_{\tau ,\langle z_{\varphi_{\tau },n_{\varphi_{\tau }}}\left(k\right)|A_{j}\left(z_{\varphi_{\tau },n_{\varphi_{\tau }}}\left(k\right)\right)\rangle }^{0}}{\prod_{\Theta }\left(1-P_{\langle z_{i}\left(k\right)|A_{j}\left(z_{i}\left(k\right)\right)\rangle }^{\sigma }\right)}. \end{array} \end{array}$$

MP-LM-IPDA applies the single target tracking structure of MP-IPDA to multitarget tracking with modulated clutter measurement densities accounting for other potential targets as well as clutter. When associating $\langle z_{\varphi_{\tau },n_{\varphi_{\tau }}}\left(k\right)|A_{j}\left(z_{\varphi_{\tau },n_{\varphi_{\tau }}}\left(k\right)\right)\rangle$ to track τ in a multitarget tracking scenario, the modulated clutter measurement density at $\langle z_{\varphi_{\tau },n_{\varphi_{\tau }}}\left(k\right)|A_{j}\left(z_{\varphi_{\tau },n_{\varphi_{\tau }}}\left(k\right)\right)\rangle$ is represented by taking into account the influence of pure clutter as well as other potential targets. This is written as

(40) $$\begin{array}{c} \begin{array}{cc} & \rho_{c,\langle z_{\varphi_{\tau },n_{\varphi_{\tau }}}\left(k\right)|A_{j}\left(z_{\varphi_{\tau },n_{\varphi_{\tau }}}\left(k\right)\right)\rangle }^{\tau }\\ = & \rho^{\varphi_{\tau }}Q_{\tau ,\langle z_{\varphi_{\tau },n_{\varphi_{\tau }}}\left(k\right)|A_{j}\left(z_{\varphi_{\tau },n_{\varphi_{\tau }}}\left(k\right)\right)\rangle }^{0}\\ & +\sum_{\forall \sigma \in T\setminus \tau }p_{\langle z_{\varphi_{\tau },n_{\varphi_{\tau }}}\left(k\right)|A_{j}\left(z_{\varphi_{\tau },n_{\varphi_{\tau }}}\left(k\right)\right)\rangle }^{\sigma }P_{\langle z_{\varphi_{\tau },n_{\varphi_{\tau }}}\left(k\right)|A_{j}\left(z_{\varphi_{\tau },n_{\varphi_{\tau }}}\left(k\right)\right)\rangle }^{\sigma }Q_{\tau ,\langle z_{\varphi_{\tau },n_{\varphi_{\tau }}}\left(k\right)|A_{j}\left(z_{\varphi_{\tau },n_{\varphi_{\tau }}}\left(k\right)\right)\rangle }^{\sigma }\\ = & Q_{\tau ,\langle z_{\varphi_{\tau },n_{\varphi_{\tau }}}\left(k\right)|A_{j}\left(z_{\varphi_{\tau },n_{\varphi_{\tau }}}\left(k\right)\right)\rangle }^{0}\left(\rho^{\varphi_{\tau }}+\sum_{\forall \sigma \in T\setminus \tau }p_{\langle z_{\varphi_{\tau },n_{\varphi_{\tau }}}\left(k\right)|A_{j}\left(z_{\varphi_{\tau },n_{\varphi_{\tau }}}\left(k\right)\right)\rangle }^{\sigma }\frac{P_{\langle z_{\varphi_{\tau },n_{\varphi_{\tau }}}\left(k\right)|A_{j}\left(z_{\varphi_{\tau },n_{\varphi_{\tau }}}\left(k\right)\right)\rangle }^{\sigma }}{\prod_{\Theta }\left(1-P_{\langle z_{i}\left(k\right)|A_{j}\left(z_{i}\left(k\right)\right)\rangle }^{\sigma }\right)}\right)\\ \overset{\Delta }{\;=} & Q_{\tau ,\langle z_{\varphi_{\tau },n_{\varphi_{\tau }}}\left(k\right)|A_{j}\left(z_{\varphi_{\tau },n_{\varphi_{\tau }}}\left(k\right)\right)\rangle }^{0}{\tilde{\rho }}_{\langle z_{\varphi_{\tau },n_{\varphi_{\tau }}}\left(k\right)|A_{j}\left(z_{\varphi_{\tau },n_{\varphi_{\tau }}}\left(k\right)\right)\rangle }^{\tau }, \end{array} \end{array}$$

in which Equations (38) and (39) are used. ${\tilde{\rho }}_{\langle z_{\varphi_{\tau },n_{\varphi_{\tau }}}\left(k\right)|A_{j}\left(z_{\varphi_{\tau },n_{\varphi_{\tau }}}\left(k\right)\right)\rangle }^{\tau }$ in (40) is defined as the modulated clutter measurement density for $\langle z_{\varphi_{\tau },n_{\varphi_{\tau }}}\left(k\right)|A_{j}\left(z_{\varphi_{\tau },n_{\varphi_{\tau }}}\left(k\right)\right)\rangle$, given as

(41) $$\begin{array}{c} \begin{array}{c} {\tilde{\rho }}_{\langle z_{\varphi_{\tau },n_{\varphi_{\tau }}}\left(k\right)|A_{j}\left(z_{\varphi_{\tau },n_{\varphi_{\tau }}}\left(k\right)\right)\rangle }^{\tau }\overset{\Delta }{\;=}\rho^{\varphi_{\tau }}+\sum_{\forall \sigma \in T\setminus \tau }\frac{p_{\langle z_{\varphi_{\tau },n_{\varphi_{\tau }}}\left(k\right)|A_{j}\left(z_{\varphi_{\tau },n_{\varphi_{\tau }}}\left(k\right)\right)\rangle }^{\sigma }P_{\langle z_{\varphi_{\tau },n_{\varphi_{\tau }}}\left(k\right)|A_{j}\left(z_{\varphi_{\tau },n_{\varphi_{\tau }}}\left(k\right)\right)\rangle }^{\sigma }}{\prod_{\Theta }\left(1-P_{\langle z_{i}\left(k\right)|A_{j}\left(z_{i}\left(k\right)\right)\rangle }^{\sigma }\right)}. \end{array} \end{array}$$

Each path pattern combined measurement cell has a unique ${\tilde{\rho }}_{\langle z_{\varphi_{\tau },n_{\varphi_{\tau }}}\left(k\right)|A_{j}\left(z_{\varphi_{\tau },n_{\varphi_{\tau }}}\left(k\right)\right)\rangle }^{\tau }$, resulting in a non-homogeneous clutter environment for the multitarget tracking.

### 3.5. MP-LM-IPDA

The calculations involved in the MP-LM-IPDA for multitarget tracking considering multi-path patterns are introduced in this section.

The clutter likelihood function $p_{k}^{c}\left(z_{k}|\chi_{k,0}^{\tau },\chi_{k}^{\tau },Z^{k-1}\right)$ in Equation (17), which considers all the obtained measurements in $Z_{k}$ as clutter, is changed to

(42) $$\begin{array}{c} \begin{array}{c} p_{k}^{c}\left(Z_{k}|\chi_{k,0}^{\tau },\chi_{k}^{\tau },Z^{k-1}\right)=\mu_{F}\left(m_{k}\right)\prod_{i=1}^{m_{k}}\frac{\rho_{c,z_{i}\left(k\right)}^{\tau }}{\lambda }, \end{array} \end{array}$$

where $\lambda =\int_{V}\rho_{c,z}^{\tau }dz=\int_{V}Q_{\tau ,z}^{0}{\tilde{\rho }}_{k,z}^{\tau }dz$ is the mean number of non-homogeneous clutter measurements in the volume *V* of the surveillance region.

For multitarget tracking, the likelihood function $p\left(Z_{k}|\chi_{k,z_{\varphi_{\tau },n_{\varphi_{\tau }}}}^{\tau },\chi_{k}^{\tau },Z^{k-1}\right)$ in Equation (16) is modified as

(43) $$\begin{array}{c} \begin{array}{c} \begin{array}{c} p\left(Z_{k}|\chi_{k,\langle z_{\varphi_{\tau },n_{\varphi_{\tau }}}\left(k\right)|A_{j}\left(z_{\varphi_{\tau },n_{\varphi_{\tau }}}\left(k\right)\right)\rangle }^{\tau },\chi_{k}^{\tau },Z^{k-1}\right)\\ =p_{\langle z_{\varphi_{\tau },n_{\varphi_{\tau }}}\left(k\right)|A_{j}\left(z_{\varphi_{\tau },n_{\varphi_{\tau }}}\left(k\right)\right)\rangle }^{\tau }Q_{\tau ,\langle z_{\varphi_{\tau },n_{\varphi_{\tau }}}\left(k\right)|A_{j}\left(z_{\varphi_{\tau },n_{\varphi_{\tau }}}\left(k\right)\right)\rangle }^{0}\frac{e^{-\lambda }}{\left(m_{k}-\varphi_{\tau }\right)!}\lambda^{m_{k}-\varphi_{\tau }}\prod_{\forall z_{i}\notin z_{\varphi_{\tau },n_{\varphi_{\tau }}}}\frac{\rho_{c,z_{i}\left(k\right)}^{\tau }}{\lambda }, \end{array} \end{array} \end{array}$$

where $p_{\langle z_{\varphi_{\tau },n_{\varphi_{\tau }}}\left(k\right)|A_{j}\left(z_{\varphi_{\tau },n_{\varphi_{\tau }}}\left(k\right)\right)\rangle }^{\tau }$ is given in (12). Note that both Equations (42) and (43) reflect the influence from other potential targets excluding target τ.

If Equations (42) and (43) are used in the derivation of MP-IPDA in place of Equations (17) and (18), respectively, the following probabilities of data associations and target existence are obtained for MP-LM-IPDA with consideration of other potential targets:

(44) $$\begin{array}{c} \begin{array}{c} \beta_{\langle z_{\varphi_{\tau },n_{\varphi_{\tau }}}\left(k\right)|A_{j}\left(z_{\varphi_{\tau },n_{\varphi_{\tau }}}\left(k\right)\right)\rangle }^{\tau }=\frac{{\left(P_{D}P_{G}\right)}^{\varphi_{\tau }}{\left(1-P_{D}P_{G}\right)}^{1-\varphi_{\tau }}}{{\tilde{\Lambda }}_{k}^{\tau }}\frac{p_{\langle z_{\varphi_{\tau },n_{\varphi_{\tau }}}\left(k\right)|A_{j}\left(z_{\varphi_{\tau },n_{\varphi_{\tau }}}\left(k\right)\right)\rangle }^{\tau }}{{\tilde{\rho }}_{\langle z_{\varphi_{\tau },n_{\varphi_{\tau }}}\left(k\right)|A_{j}\left(z_{\varphi_{\tau },n_{\varphi_{\tau }}}\left(k\right)\right)\rangle }^{\tau }} \end{array} \end{array}$$

and

(45) $$\begin{array}{c} \begin{array}{c} \beta_{k,0}^{\tau }=\frac{1-P_{Dec}^{\tau }}{{\tilde{\Lambda }}_{k}^{\tau }} \end{array} \end{array}$$

with

(46) $$\begin{array}{c} \begin{array}{c} {\tilde{\Lambda }}_{k}^{\tau }\overset{\Delta }{\;=}1-P_{Dec}^{\tau }+\sum_{\varphi_{\tau }=1}^{\varphi_{\tau ,\max}}\sum_{n_{\varphi_{\tau }}=1}^{c_{\varphi_{\tau }}}\sum_{j}\frac{p_{\langle z_{\varphi_{\tau },n_{\varphi_{\tau }}}\left(k\right)|A_{j}\left(z_{\varphi_{\tau },n_{\varphi_{\tau }}}\left(k\right)\right)\rangle }^{\tau }}{{\tilde{\rho }}_{\langle z_{\varphi_{\tau },n_{\varphi_{\tau }}}\left(k\right)|A_{j}\left(z_{\varphi_{\tau },n_{\varphi_{\tau }}}\left(k\right)\right)\rangle }^{\tau }}{\left(P_{D}P_{G}\right)}^{\varphi_{\tau }}{\left(1-P_{D}P_{G}\right)}^{L-\varphi_{\tau }}, \end{array} \end{array}$$

where ${\tilde{\rho }}_{\langle z_{\varphi_{\tau },n_{\varphi_{\tau }}}\left(k\right)|A_{j}\left(z_{\varphi_{\tau },n_{\varphi_{\tau }}}\left(k\right)\right)\rangle }^{\tau }$ is the modulated clutter measurement density at $z_{\varphi_{\tau },n_{\varphi_{\tau }}}\left(k\right)$ with path pattern $A_{j}\left(z_{\varphi_{\tau },n_{\varphi_{\tau }}}\left(k\right)\right)$, and $P_{Dec}^{\tau }$ is the probability that there is at least one target generated measurement in $Z_{k}$, as defined in (27).

The probability of target existence, in MP-LM-IPDA, is updated by

(47) $$\begin{array}{c} \begin{array}{c} P\left(\chi_{k}^{\tau }|Z^{k}\right)=\frac{{\tilde{\Lambda }}_{k}^{\tau }P\left(\chi_{k}^{\tau }|Z^{k-1}\right)}{1-\left(1-{\tilde{\Lambda }}_{k}^{\tau }\right)P\left(\chi_{k}^{\tau }|Z^{k-1}\right)}. \end{array} \end{array}$$

One can apply the extended Kalman filter (EKF) to obtain state estimates for each of the data association events [6,7], and a Gaussian mixture is used to yield the track state based on all of the data association events [20].

Compared to MP-IPDA, MP-LM-IPDA utilizes the modulated clutter measurement density instead of the pure clutter measurement density to evaluate the data association probabilities and the probability of target existence. Thus, the single target tracking structure is maintained for multitarget tracking with only a minimal additional computational load required by the calculation of ${\tilde{\rho }}_{\langle z_{\varphi_{\tau },n_{\varphi_{\tau }}}\left(k\right)|A_{j}\left(z_{\varphi_{\tau },n_{\varphi_{\tau }}}\left(k\right)\right)\rangle }^{\tau }$. The use of ${\tilde{\rho }}_{\langle z_{\varphi_{\tau },n_{\varphi_{\tau }}}\left(k\right)|A_{j}\left(z_{\varphi_{\tau },n_{\varphi_{\tau }}}\left(k\right)\right)\rangle }^{\tau }$ in MP-LM-IPDA, instead of $\rho_{\langle z_{\varphi_{\tau },n_{\varphi_{\tau }}}\left(k\right)|A_{j}\left(z_{\varphi_{\tau },n_{\varphi_{\tau }}}\left(k\right)\right)\rangle }^{\tau }$ as in a conventional tracker like MP-IPDA, when updating track τ with $\langle z_{\varphi_{\tau },n_{\varphi_{\tau }}}\left(k\right)|A_{j}\left(z_{\varphi_{\tau },n_{\varphi_{\tau }}}\left(k\right)\right)\rangle$ is the core of the proposed LM approach.

The following procedure (Algorithm 1) illustrates how to update a track in MP-LM-IPDA.

It is clear that the above procedure maintains the single target tracking structure without requiring joint assignments among the tracks and measurement cells.    

**Algorithm 1:** **MD-LM-IPDA Track Update Process**1: **for** each track τ find 2:    The track state prediction $p\left(x_{k}^{\tau }|\chi_{k}^{\tau },Z^{k-1}\right)$ and the probability of target existence prediction $P\left(\chi_{k}^{\tau }|Z^{k-1}\right)$ 3:    The measurement selection (the gating method), the measurement cell generation (the measurement partition) and the path pattern combination 4:    The modulated clutter measurement density ${\tilde{\rho }}_{\langle z_{\varphi_{\tau },n_{\varphi_{\tau }}}\left(k\right)|A_{j}\left(z_{\varphi_{\tau },n_{\varphi_{\tau }}}\left(k\right)\right)\rangle }^{\tau }$ for each path pattern combined measurement cell 5:    A posteriori data association probabilities $\beta_{\langle z_{\varphi_{\tau },n_{\varphi_{\tau }}}\left(k\right)|A_{j}\left(z_{\varphi_{\tau },n_{\varphi_{\tau }}}\left(k\right)\right)\rangle }^{\tau }$ in (44) and $\beta_{k,0}^{\tau }$ in (45) 6:    The updated probability of target existence $P\left(\chi_{k}^{\tau }|Z^{k}\right)$ in (47) 7:    The updated track state $p\left(x_{k}^{\tau }|\chi_{k}^{\tau },Z^{k}\right)$ generated by a Gaussian mixture based on all the data association events 8: **end for**

## 4. Complexity Analyses

Multi-path multitarget tracking algorithms jointly assign measurements to tracks. A detailed analysis of the complexity of joint measurements-to-track assignments is given in Section 3.3. A in [12]. As a result, the number of unique joint assignments is combinatorially increased with the number of measurements and tracks. Multi-path multitarget tracking algorithms need to enumerate and evaluate all the joint measurements-to-track assignments, which makes these algorithms hard to implement even when there is just a small number of closely spaced targets.

The MP-LM-IPDA algorithm proposed in this paper works in a completely different way. It reduces the computational load of multi-path multitarget tracking algorithms by entirely bypassing the joint assignment process. Thus, each track in MP-LM-IPDA is propagated separately. The possibility that a measurement is generated by other potential targets is evaluated in the modulated clutter measurement density. Assume that there are a total of *T* tracks and *M* path pattern combined measurement cells, the computational complexity of MP-LM-IPDA, in this case, is T×M. In effect, MP-LM-IPDA can be treated as a bank of coupled MP-IPDA filters, and the coupling is realized by the modulated clutter measurement density.

## 5. Simulation

Two simulation scenarios were considered. Compared to Scenario 1, Scenario 2 considered more targets and a higher clutter density environment. From the simulation results, MP-LM-IPDA provides a satisfactory trade-off between the implementation complexity and the tracking performance.

The simulation studies were used to compare MP-LM-IPDA with MP-JIPDA [12] and single path LM-IPDA (SP-LM-IPDA) with respect to the true track confirmation, the target state estimation accuracy, and the computational efficiency in multitarget crossing environments with clutter. Note that only one specific path (Path 1 with measurement model EE) is applied in SP-LM-IPDA, indicating that there is at most one detection from a target at each scan.

### 5.1. Simulation Scenario 1

**Figure 3:**
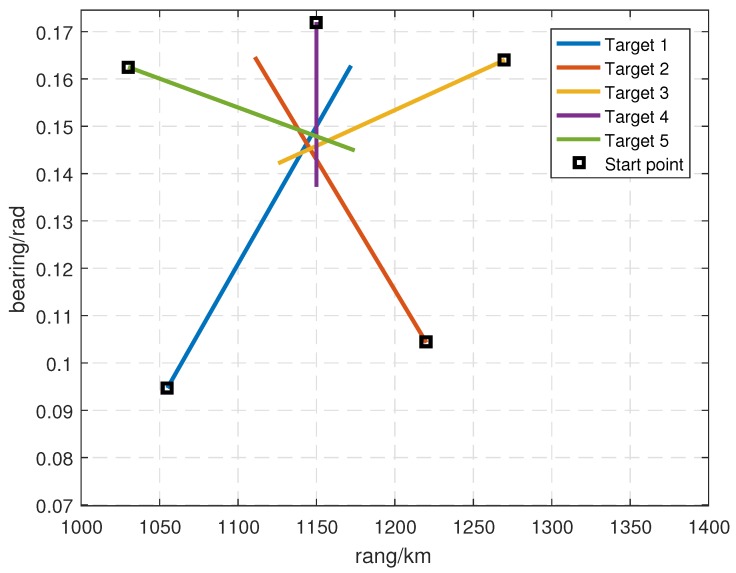
Simulation Scenario 1.

We modeled the target dynamics in the ground plane, as shown in Figure 1, using a nearly constant velocity (NCV) model given by Equation (1). As shown in Figure 3, five targets occurred at scan k=1 and disappeared at scan k=40. From scan k=30 to scan k=33, these five targets were closely spaced, which increased the complexity of the multitarget data association. The initial target states are given by

(48) $$\begin{array}{c} \mathrm{Target}\;1=\left[\begin{array}{cccc} 1055km & 0.15km/s & 0.09472rad & 8.72665\times 10^{-5}rad/s \end{array}\right]\\ \mathrm{Target}\;2=\left[\begin{array}{cccc} 1220km & -0.14km/s & 0.10432rad & 7.72665\times 10^{-5}rad/s \end{array}\right]\\ \mathrm{Target}\;3=\left[\begin{array}{cccc} 1270km & -0.185km/s & 0.16401rad & -2.79865\times 10^{-5}rad/s \end{array}\right]\\ \mathrm{Target}\;4=\left[\begin{array}{cccc} 1150km & 0km/s & 0.17201rad & -4.45665\times 10^{-5}rad/s \end{array}\right]\\ \mathrm{Target}\;5=\left[\begin{array}{cccc} 1030km & 0.185km/s & 0.16251rad & -2.25665\times 10^{-5}rad/s \end{array}\right]. \end{array}$$

The state propagation matrix *F* and the process noise covariance *Q* are

(49) $$F=\left[\begin{array}{cccc} 1 & T & 0 & 0\\ 0 & 1 & 0 & 0\\ 0 & 0 & 1 & T\\ 0 & 0 & 0 & 1 \end{array}\right]$$

with the time interval T=20 s, and

(50) $$Q=blockdiag\left(\left[\begin{array}{c} 7.8\times 10^{-1},4.4\times 10^{-4}\\ 4.4\times 10^{-4},1.3\times 10^{-5} \end{array}\right],\left[\begin{array}{c} 1.5\times 10^{-12},1.1\times 10^{-13}\\ 1.1\times 10^{-13},1.1\times 10^{-14} \end{array}\right]\right).$$

Simulation data for 200 Monte Carlo runs were tested, with each run comprising 40 scans. The measurement generation model is introduced in Section 2. The other parameters related to the simulation environment are given in Table 2, in which some parameters are similar to those given in [30,31].

Two point differencing [26] was used for track initialization and each new born track was assigned an initial PTE. The initial track state covariance is given by

(51) $$P_{0|0}=diag\left(25km^{2},1\times 10^{-5}km^{2}/s^{2},9\times 10^{-6}rad^{2},6.4\times 10^{-8}rad^{2}/s^{2}\right).$$

The parameters used for the predicted probability of target existence calculation are

(52) $$a_{11}=0.98\;and\;a_{21}=0,$$

in which $a_{21}$ was set 0 since the track birth was considered by the track initialization process.

Different tracking algorithms were compared such that they had the same number of confirmed false tracks controlled by utilizing different values of the “initial PTE” and maintaining the same value of the “confirmation PTE”, as depicted in Table 3.

**Figure 4:**
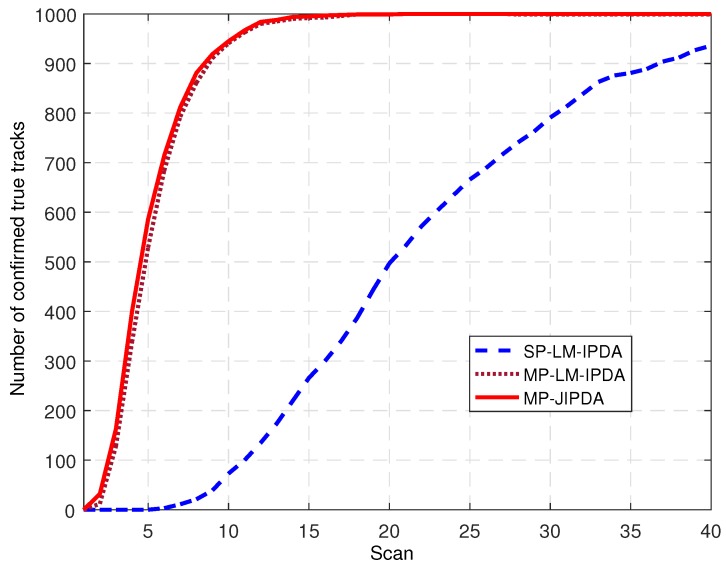
Number of confirmed true tracks for all targets.

**Figure 5:**
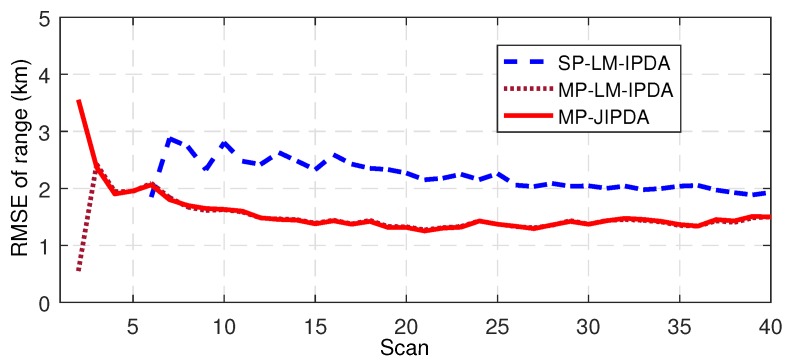
RMSE of range for Target 1.

**Figure 6:**
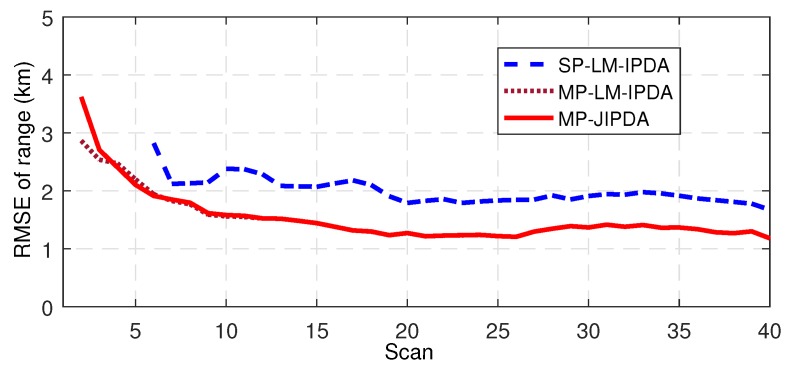
RMSE of range for Target 3.

**Figure 7:**
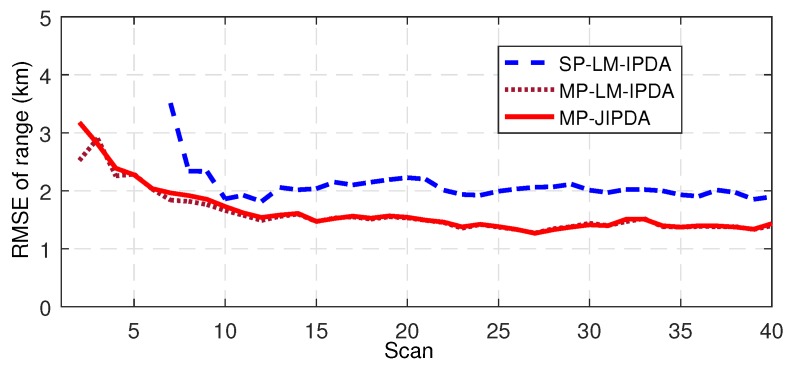
RMSE of range for Target 5.

**Figure 8:**
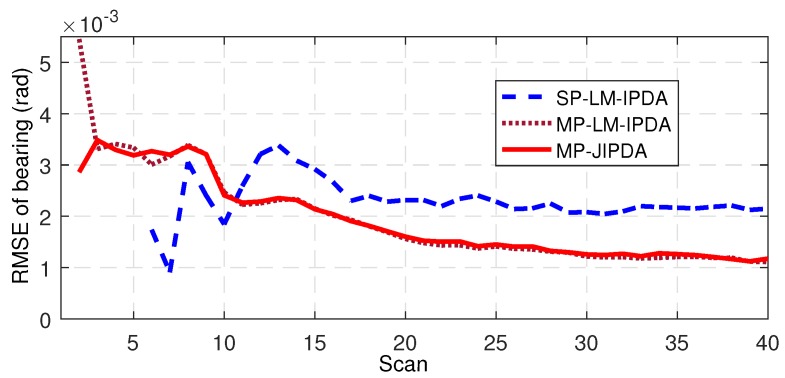
RMSE of bearing for Target 1.

**Figure 9:**
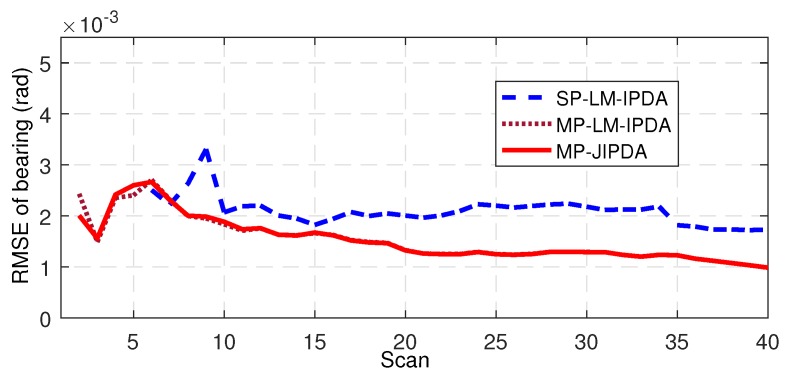
RMSE of bearing for Target 3.

**Figure 10:**
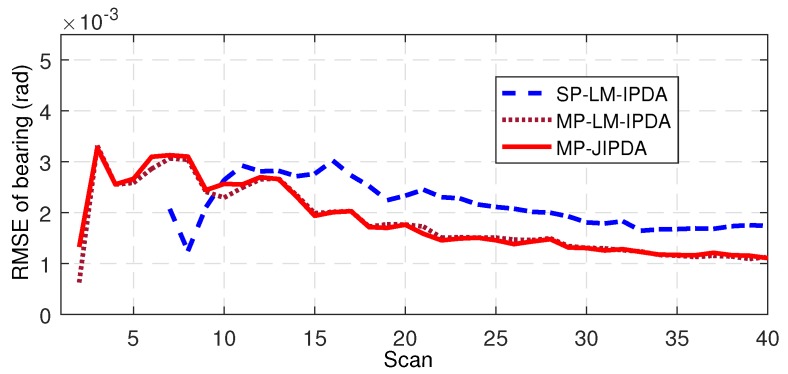
RMSE of bearing for Target 5.

The number of confirmed true tracks, the RMSE of the range, and the RMSE of the bearing are shown in Figure 4, Figure 5, Figure 6, Figure 7, Figure 8, Figure 9 and Figure 10. The detailed process of generating the number of confirmed true tracks is introduced in [12,20].

The number of confirmed true tracks (CTTs) following each of the five targets for 200 runs is depicted in Figure 4. This statistics parameter was used to record the number of tracks that were following targets at each scan. In Figure 4, the perfect number of confirmed true tracks (i.e., 100%) is 1000. In Figure 4, we can see that MP-LM-IPDA and MP-JIPDA had excellent true track confirmation performances where the numbers of CTTs increased to the maximum value (1000) after an initial period of time and then remained stable, even around the target intersect scans. In the initial period of time, MP-JIPDA had more CTTs than MP-LM-IPDA, for example at scan k=6, the numbers of CTTs of MP-JIPDA and MP-LM-IPDA were 714 and 684, respectively. However, the performance of SP-LM-IPDA was unsatisfactory in that the number of the CTTs grew slowly and could not attain the maximum value. The performances shown in Figure 4 indicate the multi-path pattern provided sufficient information on the true track confirmation, thus the multi-path algorithms (MP-LM-IPDA and MP-JIPDA) achieved much better performances compared to the single path algorithm (SP-LM-IPDA).

For the tracking accuracy evaluation, the RMSEs of Targets 1, 3 and 5 are demonstrated here. Figure 5, Figure 6, Figure 7, Figure 8, Figure 9 and Figure 10 depict the RMSEs for the range estimation and bearing estimation. All three algorithms had the same trend in that the estimation errors decreased as the scan increased due to more target state information being collected. In addition, the performances of MP-LM-IPDA and MP-JIPDA were overall significantly better than that of SP-LM-IPDA because of sufficient handling of the multi-path dependent target information. MP-LM-IPDA obtained almost the same track accuracy as MP-JIPDA.

The simulation was implemented on a 4.00 GHz, Intel Core i7 PC and run with MATLAB. The CPU times per each Monte Carlo run of SP-LM-IPDA, MP-LM-IPDA and MP-JIPDA were 2.63 s, 6.46 s and 21.44 s, respectively.

### 5.2. Simulation Scenario 2

**Figure 11:**
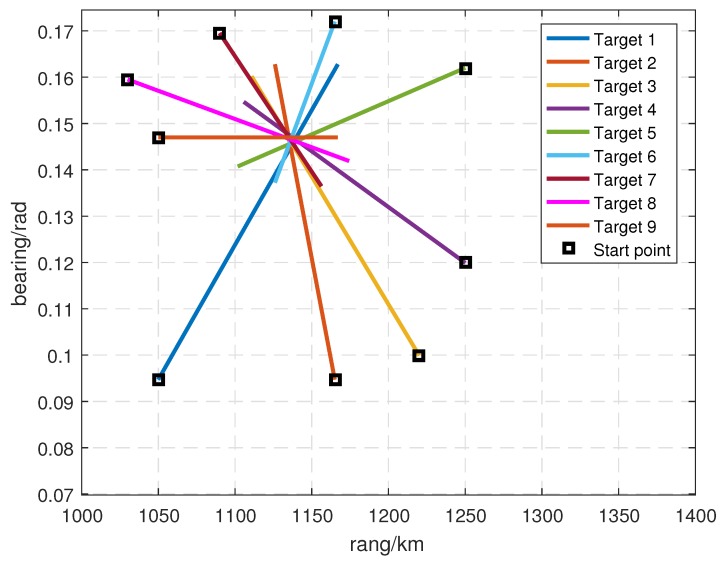
Simulation Scenario 2.

Compared to Scenario 1, there were nine targets in this scenario, as shown in Figure 11. Moreover, the mean number of clutter per each scan was increased to 50. Therefore, the tracking task was more burdensome as there could be more measurements shared among tracks, which leads to heavier data association complexity.

These nine targets appeared at scan k=1, disappeared at scan k=40, and were most closely spaced around scan k=30. Trajectories of these nine targets are shown in Figure 11, and the initial states of these nine targets are given by

(53) $$\begin{array}{c} \mathrm{Target}\;1=\left[\begin{array}{cccc} 1050km & 0.15km/s & 0.09472rad & 8.72665\times 10^{-5}rad/s \end{array}\right]\\ \mathrm{Target}\;2=\left[\begin{array}{cccc} 1165km & -0.05km/s & 0.09472rad & 8.72665\times 10^{-5}rad/s \end{array}\right]\\ \mathrm{Target}\;3=\left[\begin{array}{cccc} 1220km & -0.14km/s & 0.09992rad & 7.72665\times 10^{-5}rad/s \end{array}\right]\\ \mathrm{Target}\;4=\left[\begin{array}{cccc} 1250km & -0.185km/s & 0.11992rad & 4.45665\times 10^{-5}rad/s \end{array}\right]\\ \mathrm{Target}\;5=\left[\begin{array}{cccc} 1250km & -0.19km/s & 0.16201rad & -2.72665\times 10^{-5}rad/s \end{array}\right]\\ \mathrm{Target}\;6=\left[\begin{array}{cccc} 1165km & -0.05km/s & 0.17201rad & -4.45665\times 10^{-5}rad/s \end{array}\right]\\ \mathrm{Target}\;7=\left[\begin{array}{cccc} 1090km & 0.085km/s & 0.16951rad & -4.23665\times 10^{-5}rad/s \end{array}\right]\\ \mathrm{Target}\;8=\left[\begin{array}{cccc} 1030km & 0.185km/s & 0.15951rad & -2.25665\times 10^{-5}rad/s \end{array}\right]\\ \mathrm{Target}\;9=\left[\begin{array}{cccc} 1050km & 0.15km/s & 0.14701rad & 0rad/s \end{array}\right] \end{array}$$

SP-LM-IPDA, MP-LM-IPDA and MP-JIPDA were compared such that they had the same number of confirmed false tracks by utilizing different values of the “initial PTE” and maintaining the same value of the “confirmation PTE”, as depicted in Table 4. The values of other simulation parameters are the same as those given in the Scenario 1.

**Figure 12:**
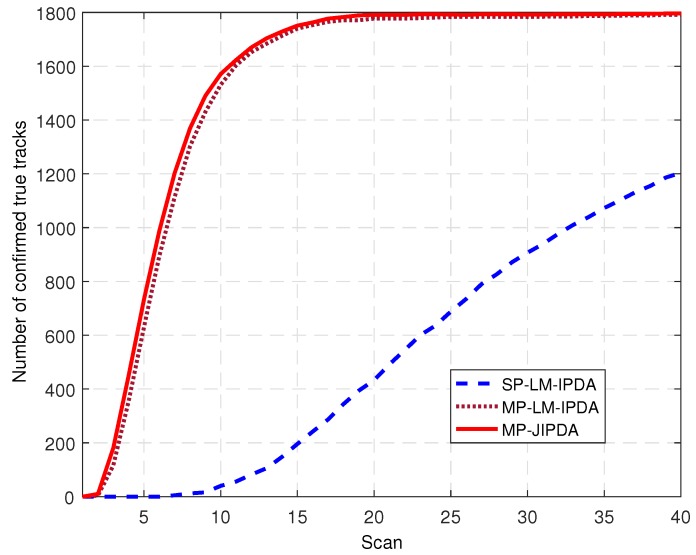
Number of confirmed true tracks for all targets.

**Figure 13:**
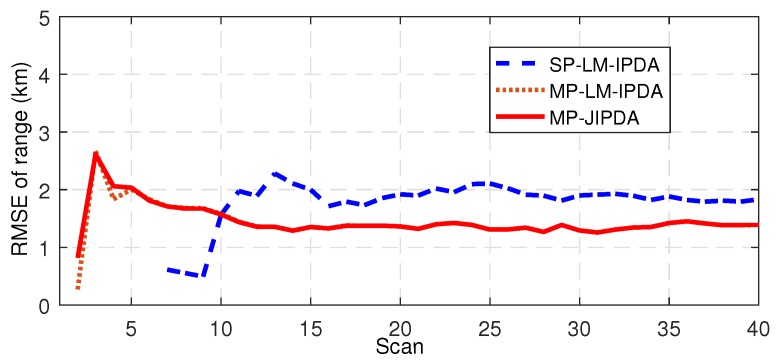
RMSE of range for Target 3.

**Figure 14:**
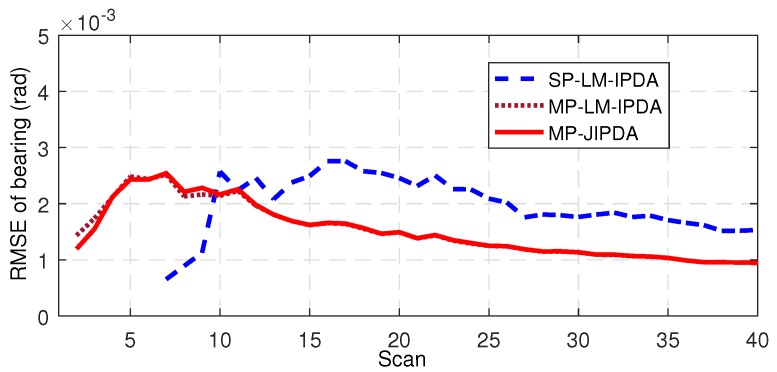
RMSE of bearing for Target 3.

The number of the confirmed true tracks, the RMSE of the range, and the RMSE of the bearing are shown in Figure 12, Figure 13 and Figure 14.

The CTTs following each of the nine targets for 200 runs is depicted in Figure 12. In this figure, the perfect number of CTTs (i.e., 100%) is 1800. In this figure, we can see that the increasing rates of the CTTs of all the three algorithms were reduced compared to the performances shown in Figure 4. This is because there were more clutter measurements. When the numbers of CTTs of MP-LM-IPDA and MP-JIPDA reached a stable level, the maximum values were 1791 and 1796, respectively. The performance of SP-LM-IPDA became even worse compared to MP-LM-IPDA and MP-JIPDA in this scenario.

Here, we use the RMSE of Target 3 as an example to show the performance of the tracking accuracy. As shown in Figure 13 and Figure 14, the performances of MP-LM-IPDA and MP-JIPDA were almost the same, and both MP-LM-IPDA and MP-JIPDA had much better performances compared to SP-LM-IPDA.

In this scenario, the CPU times per each Monte Carlo run of SP-LM-IPDA, MP-LM-IPDA and MP-JIPDA were 5.97 s, 17.72 s and 117.54 s, respectively.

Both simulations demonstrate the effectiveness of MP-LM-IPDA, which had comparably good performances regarding true track confirmation and target state estimation, through just a fraction of the execution time required for MP-JIPDA. In Scenario 1, MP-LM-IPDA costs 30% of the execution time for MP-JIPDA. In Scenario 2, MP-LM-IPDA took only 15% of the execution time of MP-JIPDA. Thus, MP-LM-IPDA was more efficient compared to MP-JIPDA, especially in the target crossing scenario in a highly dense cluttered environment. Comparing the results of these two simulation scenarios, more closely spaced targets in a highly dense cluttered environment created heavier data association burden for all algorithms, resulting in the true track confirmation performance degeneration and the computational time increase.

## 6. Conclusions

In this paper, MP-LM-IPDA is proposed to resolve the measurement origin uncertainty and the measurement path uncertainty problems for multitarget tracking with the OTHR system. The main benefit of this approach is that it bypasses the generation of feasible joint events among the tracks, the measurements, and the paths. The modulated clutter measurement density, specified by each path pattern combined measurement cell, is introduced for multitarget tracking. Utilizing the modulated clutter measurement density, each track propagates separately, i.e., the single target tracking structure could be applied for multitarget tracking.

To demonstrate the superiority of the proposed algorithm, we compared it with the MP-JIPDA algorithm and the SP-LM-IPDA algorithm in two multitarget tracking environments using OTHR measurements. The simulation results indicate that this new algorithm provides a trade-off between the computational complexity and the performance. Based on the results, the proposed algorithm could be considered as one of the efficient approaches in multitarget tracking for OTHR, especially tracking in complex situations where a large number of closely spaced targets are involved in highly dense cluttered environments.

Our future work will consider time-varying ionospheric layer heights in MP-LM-IPDA, geared toward making the proposed algorithm more suitable for practical use.

## Figures and Tables

**Table 1:** Signal propagation paths.

Path	Model	Transmit Layer $h_{t}$	Receive Layer $h_{r}$
1	EE	$h_{E}$	$h_{E}$
2	EF	$h_{E}$	$h_{F}$
3	FE	$h_{F}$	$h_{E}$
4	FF	$h_{F}$	$h_{F}$

**Table 2:** Simulation parameters.

Parameter	Value
Slant range size	1000–1400 km
Rate of slant range size	0.013889–0.22222 km/s
Apparent azimuth size	0.069813–0.17453 rad
Mean number of clutter per each scan	25
Transmitter to receiver distance *d*	100 km
Hight of layer *E*, $h_{E}$	100 km
Hight of layer *F*, $h_{F}$	260 km
Target detection probability in each path	$P_{D}$ = 0.4
Gating probability	$P_{G}$ = 0.997
Measurement noise covariance *R*	diag (25 km${}^{2}$, 1 × 10${}^{-6}$ km${}^{2}$/s${}^{2}$, 9 × 10${}^{-6}$ rad${}^{2}$)

**Table 3:** Simulation parameters for different algorithms (Scenario 1).

	SP-LM-IPDA	MP-LM-IPDA	MP-JIPDA
Initial PTE	0.000093	0.0009	0.0025
Confirmation PTE	0.98	0.98	0.98
Termination PTE	0.000093/5	0.0009/5	0.0025/5
Number of CFTs	5	5	5

**Table 4:** Simulation parameters for different algorithms (Scenario 2).

	SP-LM-IPDA	MP-LM-IPDA	MP-JIPDA
Initial PTE	0.00008	0.0009	0.0027
Confirmation PTE	0.98	0.98	0.98
Termination PTE	0.00008/5	0.0009/5	0.0027/5
Number of CFTs	7	7	7

## Abbreviations

The following abbreviations are used in this manuscript:

PDA	Probabilistic data association.
MD-JPDA	Multiple detection joint probabilistic data association.
MHT	Multiple hypothesis tracker.
LM-IPDA	Linear multitarget integrated probabilistic data association.
PTE	Probability of target existence.
OTHR	Over-the-horizon-radar.
MP-JIPDA	Multi-path joint integrated probabilistic data association.
MP-LM-IPDA	Multi-path LM-IPDA.
NCV	Nearly constant velocity.
CFT	Confirmed false track.
CTTs	Number of the confirmed true tracks.
RMSE	Root mean square error.
Nomenclature	
τ	A track as well as the potential target being tracked by this track.
$m_{k}$	The number of selected measurements at scan *k*.
*L*	The number of signal propagation paths in the OTHR system.
$\varphi_{\tau ,\max}$	The maximum number of target-originated measurements, which satisfies $\varphi_{\tau ,\max}=\min\left(L,m_{k}\right)$.
$\varphi_{\tau }$	The number of target originated measurements $\varphi_{\tau }\in \left\{1,2,\ldots ,\varphi_{\tau ,\max}\right\}$.
$n_{\varphi_{\tau }}$	A variable that enumerates the measurement cells under the condition that there are $\varphi_{\tau }$ measurements generated by target τ, $n_{\varphi_{\tau }}\in \left\{1,2,\ldots ,c_{\varphi_{\tau }}\right\}$ and $c_{\varphi_{\tau }}=C_{\varphi_{\tau }}^{m_{k}}=\frac{m_{k}!}{\varphi_{\tau }!\left(m_{k}-\varphi_{\tau }\right)!}$.
$z_{\varphi_{\tau },n_{\varphi_{\tau }}}\left(k\right)$	A measurement cell specified by $\varphi_{\tau }$ and $n_{\varphi_{\tau }}$ at scan *k*.
$\chi_{k}^{\tau }$	The event that target τ exists at scan *k*.
